# Progress in the application of the neutrophil-to-lymphocyte ratio in dialysis-related complications

**DOI:** 10.1080/0886022X.2023.2259996

**Published:** 2023-10-04

**Authors:** Nan Yang, Kaibi Yang, Shujun Pan, Qiang He, Juan Jin

**Affiliations:** Department of Nephrology, The First Affiliated Hospital of Zhejiang Chinese Medical University (Zhejiang Provincial Hospital of Traditional Chinese Medicine), Hangzhou, Zhejiang, China

**Keywords:** Neutrophil-to-lymphocyte ratio, hemodialysis, peritoneal dialysis, complications, disease burden

## Abstract

The neutrophil-to-lymphocyte ratio (NLR) is a novel predictive biomarker that reflects systemic inflammatory status and is routinely measured in blood tests. Owing to its ease of use and affordability, it is being increasing used as a prognostic indicator of cardiovascular disease, tumors, autoimmune disorders, and kidney disease. In recent years, a number of studies have demonstrated the clinical utility of the NLR in identifying and predicting complications associated with hemodialysis and peritoneal dialysis, including cardiovascular disease and infection. This review aimed to provide a new perspective on the application of the NLR as a valuable tool enabling clinicians to better assess the occurrence and prognosis of complications in patients undergoing dialysis.

## Introduction

The neutrophil-to-lymphocyte ratio (NLR) is a novel biomarker of systemic inflammation. Neutrophils are the primary responders to cellular or tissue infections and as such are a crucial component of the innate immune system. They are recruited to the inflammatory site by cytokines and chemokines, and mount either an anti-inflammatory or a proinflammatory immune response depending on the degree of inflammation [1]. Neutrophils also play a vital role in adaptive immunity and are the principal effector cells in systemic inflammatory response syndrome. As key regulators of innate immunity, neutrophils recruit, activate, and orchestrate other immune cells, including dendritic cells, CD4^+^ T cells, and CD8^+^ T-cells, through the secretion of a diverse array of cytokines and chemokines [2]. Neutrophil counts are typically increased in acute infection, myocardial infarction, severe trauma, or postoperative complications. Lymphocytes, the smallest white blood cells, are another integral component of the immune system that play a pivotal role in safeguarding against external infections and monitoring internal cellular variations. Lymphocytes possess immune recognition capabilities and can be categorized into T, B, and natural killer cells. Among these subsets, T and B cells are antigen-specific lymphocytes originating from hematopoietic tissue [3]. Neutrophils and lymphocytes can be routinely detected in blood tests without the need for additional samples, thereby reducing experimental costs and improving patient satisfaction. Although C-reactive protein (CRP) is widely acknowledged as the gold standard inflammatory indicator, it is not included as a routine test item. Similarly, other inflammatory markers, such as interleukin-6 and tumor necrosis factor, are not commonly part of routine testing. Previous studies have demonstrated a positive correlation between the NLR and CRP levels, indicating that patients with elevated levels of inflammation exhibit higher NLRs [4,5]. Owing to the simple acquisition method, low cost, and widespread acceptance of the NLR, researchers have begun to explore its potential applications in various diseases, such as cardiovascular disease [6], tumors [7], and kidney disease [8].

**Figure 1.**
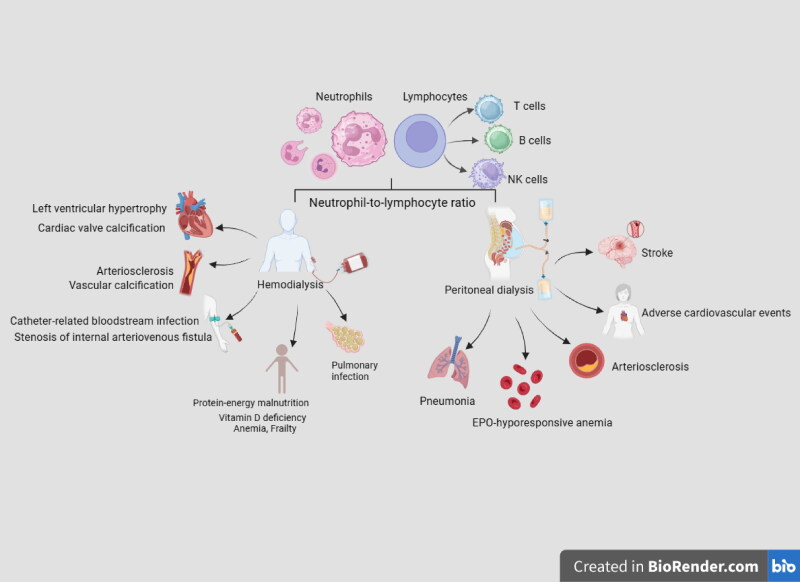
Diagram of potential clinical applications of the neutrophil-to-lymphocyte ratio in the diagnosis and prognosis of complications associated with hemodialysis and peritoneal dialysis. EPO, erythropoietin; NK cells, natural killer cells.

Patients with advanced kidney disease primarily opt for dialysis as a renal replacement therapy. There are two types of dialysis, hemodialysis (HD) and peritoneal dialysis (PD); both methods extend the lifespan of patients, but both may also give rise to certain complications such as cardiovascular events, infections, and even mortality. The prompt identification and treatment of dialysis-related complications is vital. In the advanced stages of chronic kidney disease, a systemic microinflammatory state exists in the body. Some studies have indicated that neither HD nor PD can alleviate this inflammatory state [9]. The NLR integrates information regarding two distinct leukocyte subtypes for a more precise assessment of the immune response and superior clinical significance compared with other inflammatory markers. The NLR has been shown to be significantly associated with inflammation and mortality [10,11], and can predict mortality in patients undergoing dialysis [12]. Therefore, an increasing number of studies are exploring the clinical significance of the NLR in complications arising from HD (Table 1, Figure 1) and PD (Table 2, Figure 1).

**Table 1.** Application of the neutrophil to lymphocyte ratio in hemodialysis-related complications.

Area	Related complications	Significance of NLR	Reference
Cardiovascular	Left ventricular hypertrophy	Increased NLR level predicted left ventricular hypertrophy. NLR ≥3.5 was strongly associated with an increased risk of all-cause death and cardiovascular death.	[17]
	Vascular calcification	High NLR indicated coronary artery calcification and thoracic aortic calcification.	[15]
	Cardiac valve calcification	Degree of heart valve calcification was positively correlated with the NLR.	[19]
	Arteriosclerosis	Low NLR was associated with higher low-density lipoprotein levels. NLR predicted arterial stiffness.	[23,29]
Infection	Catheter-related bloodstream infection	NLR had a certain value in the diagnosis of catheter-related bloodstream infection.	[33,34]
	Pulmonary infection	NLR was an independent risk factor of pulmonary infection. NLR was positively correlated with disease severity and mortality in patients with coronavirus disease 2019.	[36,38]
Other	Protein-energy malnutrition	NLR was negatively correlated with serum albumin levels. There was a significant direct correlation between baseline NLR and body mass index.	[44]
	Stenosis of internal arteriovenous fistula	NLR was an independent predictor of arteriovenous fistula stenosis. Elevated NLR may be a risk factor for early AVF restenosis after successful percutaneous transluminal angioplasty.	[45,46]
	Vitamin D deficiency	Significant negative correlation between NLR and serum vitamin D levels; an elevated NLR typically indicated vitamin D insufficiency.	[48]
	Anemia	High NLR predicted poor erythropoietin responsiveness.	[49]
	Frailty	NLR was independently associated with frailty and had a certain diagnostic value for frailty.	[51]

NLR, neutrophil-to-lymphocyte ratio.

**Table 2.** Application of the neutrophil to lymphocyte ratio in peritoneal dialysis-related complications.

Area	Related complications	Significance of NLR	Reference
Cardiovascular	Adverse cardiovascular events	Increased NLR was predictive of adverse cardiovascular events in patients <60 years. Compared with patients with a high NLR, those with a low NLR had a higher survival rate. High NLR was an independent prognostic indicator of all-cause mortality. Increased NLR was significantly associated with increased all-cause mortality.	[56–58]
	Left ventricular systolic dysfunction	NLR >3.6 was significantly associated with left ventricular systolic dysfunction.	[59]
	Arteriosclerosis	NLR was independently associated with brachial-ankle pulse wave velocity and was an independent risk factor for increased arterial stiffness.	[61]
	Stroke	High NLR was an independent risk factor for first stroke.	[65]
Infection	Peritoneal dialysis-associated peritonitis	A higher NLR at the onset of peritoneal dialysis-associated peritonitis was linearly associated with an increased risk of treatment failure.	[67]
	Pneumonia	Incidence of first pneumonia was greater in the high NLR group than that in the low NLR group; the risk of pneumonia in patients with a high NLR was significantly increased.	[71]
Other	Mortality	Elevated NLR was a significant risk factor for early death, NLR >3.71 indicated a lower survival rate.	[73]
	EPO-hyporesponsive anemia	High NLR was associated with a non-significantly increased erythropoietin resistance index, but no direct correlation was established.	[5]

NLR, neutrophil-to-lymphocyte ratio.

## NLR and HD

### NLR and cardiovascular events

The mortality rate of patients undergoing HD is higher than that of the general population, with cardiovascular events being a major cause of death. Moreover, the risk factors for cardiovascular events in patients undergoing HD differ from those in the general population [13]. During the early stages of HD, alterations in the organization and function of the heart and large blood vessels increase the risk of cardiovascular complications. Over time, the heart undergoes adaptive changes, including left ventricular hypertrophy and expansion, which may result in systolic and diastolic dysfunction [14]. Calcification can also occur in the coronary arteries, thoracic aorta, and cardiac valves [15].

Left ventricular hypertrophy is an independent predictor of cardiovascular events [16] and can be diagnosed using the left ventricular mass index (LVMI). In recent years, the value of the NLR in predicting left ventricular hypertrophy in patients undergoing dialysis has been investigated. Li et al. [17] followed-up 268 patients receiving HD for 36 months. A high NLR was associated with an increased LVMI (*r* = 0.566; *p* < 0.01) and intima-media thickness (*r* = 0.578). An NLR ≥3.5 was strongly associated with a heightened risk of all-cause mortality and cardiovascular mortality; therefore the NLR has significant prognostic value for assessing cardiovascular risk and mortality in patients undergoing HD.

Coronary artery and thoracic aorta calcification are commonly used indicators of vascular calcification in patients undergoing HD. A cross-sectional study of 90 patients with end-stage renal disease undergoing HD or PD found a positive correlation between the NLR and thoracic aortic calcification score (rs = 0.334, *p* < 0.01). This suggests that a high NLR may potentially be an effective indicator of vascular calcification in patients undergoing HD^15^.

Cardiac valve calcification is a major contributor to cardiovascular events in patients receiving HD, with aortic and mitral valve calcification occurring in 25–59% of patients undergoing dialysis [18]. The etiology of cardiac valve calcification is multifactorial and involves genetics, mechanical stress, metabolic dysfunction, inflammation, mineral imbalances, and drug effects [18]. Some studies have found a close relationship between the NLR and the severity of cardiac valve calcification. A study involving 80 patients receiving HD found that the NLR was significantly higher in patients with cardiac valve calcification than that in those without (*p* < 0.05). Moreover, within the cardiac valve calcification group, patients with severe calcification had a higher NLR than those with moderate or mild calcification (*p* < 0.05); the degree of cardiac valve calcification was positively correlated with the NLR (*p* < 0.05) [19]. This study has established a basis for future clinical prediction and diagnosis of valve calcification in patients undergoing HD. Studies by Zhu et al. [20], Neuen et al. [21] and Lano et al. [22] confirmed these findings, showing that the higher the NLR, the greater the risk of cardiovascular events and mortality in patients undergoing dialysis. However, a low NLR in patients receiving HD has been associated with high levels of low-density lipoprotein, suggesting that these patients may be at an increased risk of cardiovascular disease [23]. These contradictory findings may be related to the nutritional status and serum albumin levels of patients, as malnutrition and inflammation are both associated with increased all-cause and cardiovascular mortality in patients undergoing HD. Therefore, to investigate the potential use of the NLR, it is imperative to conduct nutritional index assessments in study participants [24]. Future investigations involving larger sample sizes are warranted to validate the impact of the NLR on cardiovascular events in patients undergoing dialysis.

Increased arterial stiffness is a significant predictor of mortality in patients undergoing dialysis [25]. It is a robust predictor of increased cardiovascular risk and an early indicator of structural and functional vessel wall alterations [26]. Pulse-wave velocity (PWV) is a noninvasive and reliable measure that effectively detects arterial stiffness, as this condition leads to a decrease in arterial buffering capacity and an increase in pulse pressure and PWV. Owing to the mild inflammation that occurs during the development of arteriosclerosis, which contributes to disease progression, this condition is classified as an inflammatory disease [27,28]. The NLR has been shown to be associated with arterial stiffness and PWV^29^. Inflammation and arterial stiffness serve as predictive factors for cardiovascular events, and a comprehensive understanding of their underlying mechanisms will facilitate the selection of appropriate anti-inflammatory interventions, thereby mitigating cardiovascular risk in patients undergoing dialysis [30].

In summary, the frequent occurrence of cardiovascular events is an important cause of increased mortality in patients undergoing dialysis. Therefore, close attention should be paid to cardiovascular events in these patients. The NLR is anticipated to emerge as a novel prognostic indicator of the occurrence, progression, and prognosis of cardiovascular events in patients undergoing HD.

### NLR and infection

Infection is a pervasive complication of dialysis and a significant cause of hospitalization, morbidity, and mortality in patients undergoing HD due to their compromised immune function. Catheter-related bloodstream infections are common in these patients [31]. Previous studies have indicated that *Staphylococcus aureus* is the most common cause of infection in patients receiving HD, with vascular access infection rates ranging from 27.7% to 50% [32]. Severe bloodstream infections are associated with increased mortality [33]. A retrospective study involving 296 patients receiving HD showed that the NLR had a certain diagnostic value in catheter-related bloodstream infections, with an optimal NLR cutoff value of 4.485; however, this value was limited by low sensitivity and specificity [34].

In clinical practice, pulmonary infections are common in patients undergoing dialysis [35]; these impact the quality of life of patients, as well as being a major cause of death [36]. The NLR has been correlated with lung infection in patients undergoing HD and can be used as an effective index to evaluate pulmonary infection [36]. The NLR is an independent risk factor for pneumonia in patients undergoing HD; for every increase of 1 in the NLR, the risk of pneumonia increases by 7.2% (*p* = 0.035) [37]. A meta-analysis revealed a positive correlation between the NLR at the time of coronavirus disease 2019 (COVID-19) infection and both disease severity and mortality in adult patients with COVID-19^38^. Similar findings have been observed in individuals with chronic kidney disease who contracted COVID-19[39]. A statistically significant increase in mortality was observed in patients undergoing HD with COVID-19 when NLR thresholds were set at ≥3.0 and ≥3.5[40]. Therefore, patients undergoing HD who contract COVID-19 require additional care because of their increased risk of death. Studies have consistently demonstrated that the NLR exhibits superior sensitivity and specificity to CRP in patients undergoing dialysis with persistent microinflammation [41]. In addition, the NLR is positively correlated with the length of stay in hospital, with Lo et al. [42] reporting that the combination of the NLR and parathyroid hormone levels is a significant determinant for predicting longer hospital stays (*p* < 0.01) in patients with non-traumatic acute abdomen who are undergoing dialysis. The infection burden increases in patients undergoing dialysis and preventing and eliminating infections in these patients is a crucial responsibility of dialysis centers. It is imperative that both patients and medical staff effectively identify infections and intervene promptly. The NLR may be a valuable tool in this context, in addition to conventional inflammatory indicators.

### NLR and other complications

Coexisting protein-energy malnutrition and inflammation is frequently observed in patients undergoing maintenance dialysis. Early detection and effective management of malnutrition-inflammation complex syndrome has the potential to improve prognosis in individuals undergoing dialysis [43]. A prospective cohort study involving 77 participants undergoing HD revealed a significant inverse association between the NLR and albumin levels (*p* < 0.05). Patients with an NLR ≤1.75 had both lower hospitalization rates and a survival rate of 100% (*p* < 0.05), and a low baseline NLR was predictive of reduced hospitalization risk in diabetic patients undergoing HD [44].

An arteriovenous fistula (AVF) is the primary vascular access point used in patients undergoing HD. Stenosis, primarily caused by neointimal hyperplasia, is the most common complication associated with the long-term use of an AVF; inflammation is a contributing factor. A study by Yilmaz et al. [45] found that the NLR was correlated with the degree of AVF stenosis (*r* = 0.625; *p* < 0.01). The NLR (using an optimal cutoff value of 2.70) predicted AVF stenosis with an area under the receiving operator characteristic curve of 0.893, sensitivity of 98.4%, and specificity of 75% (*p* < 0.01). These results indicate that the NLR is an independent predictor of AVF stenosis. Other studies have shown that an elevated NLR may be a risk factor for early AVF restenosis after successful percutaneous transluminal angioplasty in patients undergoing HD [46]. Furthermore, a study by Bashar et al. [47] reported a correlation between an increased NLR and fistula maturity. However, due to the limited number of studies in this field, further research is required to confirm these findings.

Long-term HD is associated with disorders in calcium and phosphorus metabolism as well as vitamin D deficiency; these are caused by a variety of factors. A cross-sectional study by Kara and Soylu [48] revealed a significant negative correlation between the NLR and serum vitamin D levels in patients undergoing HD (r=-0.219, *p* < 0.05). The mean NLR was 2.7 in patients with vitamin D deficiency and 2.4 in patients with normal vitamin D levels, suggesting that an elevated NLR indicates inadequate vitamin D levels in patients receiving HD. Due to the relatively inadequate production of erythropoietin (EPO) in patients with chronic kidney disease, anemia is highly prevalent in this population. Consequently, EPO has received significant attention for its potential to enhance patients’ quality of life. However, conditions such as inflammation and malnutrition can reduce the efficacy of EPO treatment. The EPO resistance index can therefore serve as a valuable biomarker for predicting the risk of all-cause mortality in patients undergoing HD. Studies have shown a significant correlation between the NLR and EPO resistance index, and the NLR is considered superior to neutrophil count for the prediction of EPO resistance. At NLR >3.34, the EPO resistance index increased significantly, along with resistance to EPO[49]. This finding was substantiated in a study by Valga et al. [50]

Patients undergoing HD frequently exhibit a diverse array of symptoms attributed to urotoxins, including depression, weakness, and fatigue. These impose a significant burden on patients, necessitating vigilant attention and prompt implementation of appropriate therapeutic interventions. Frailty is characterized by the diminished capacity of the body to effectively respond to external stimuli. A study conducted by Wang et al. [51] demonstrated that the NLR is an independent risk factor for frailty in patients undergoing HD (*p* < 0.05). An optimal cutoff value of 2.98 was determined and used to divide patients into two groups. The NLR exhibited clinical significance in diagnosing frailty, with a lower survival rate in the high NLR group than that in the low NLR group[52]. One study assessed the use of the NLR in predicting dialysis initiation; uremic symptoms were associated with a higher NLR, which significantly predicted the presence of neurological and gastrointestinal symptoms, with NLR cutoff values of 2.4 and 3.0, respectively. An NLR of 2.5 six months before dialysis initiation was associated with the presence of uremic syndrome. In addition, prior to HD initiation a higher NLR may be associated with the severity of chronic kidney disease and mineral bone disease. Therefore, the NLR can be used to predict uremic symptoms and the need for HD in patients with chronic kidney disease [53]. These results enhance our understanding of the association between the NLR and quality of life in individuals undergoing dialysis.

## NLR and PD

### NLR and cardiovascular events

Cardiovascular events also remain the leading cause of mortality in patients undergoing PD, underscoring the critical importance of early detection and prevention strategies in these patients [54,55]. Despite this pressing need, there is a lack of research examining adverse cardiovascular outcomes and the NLR in patients undergoing PD. Recently, a retrospective study conducted in China recruited 1652 patients to investigate the association between adverse cardiovascular events and the NLR in patients receiving PD [56]. The cumulative incidence curve demonstrated significant differences in new cardiovascular events among different NLR groups (*p* < 0.01). Additionally, the study revealed a correlation between an elevated NLR and adverse cardiovascular event prognosis in patients under 60 years of age receiving PD [56]. The NLR may therefore be a novel predictive biomarker for assessing the risk of adverse cardiovascular events in patients undergoing PD. The NLR has also been demonstrated to be a robust prognostic indicator for long-term outcomes in patients undergoing PD; patients with a lower NLR exhibit a higher survival rate than those with a higher NLR, and an elevated NLR serves as an independent indicator of increased all-cause mortality [57]. Another study demonstrated that a persistently elevated NLR was significantly associated with increased all-cause mortality in patients undergoing PD [57,58]. Furthermore, regular and continuous NLR monitoring enabled early identification of patients at risk for adverse outcomes. The NLR has also been associated with left ventricular systolic dysfunction [59] and cardiovascular mortality (hazard ratio 2.886, 95% confidence interval 1.005–8.283, *p* < 0.05) in patients undergoing PD, and was a reliable predictor of long-term prognosis [57].

Patients undergoing PD experience persistent inflammation, resulting in increased arterial stiffness [60]. A cross-sectional study by Cai et al. [61] examined the association between the NLR and arterial stiffness, as measured by brachial-ankle PWV (BAPWV) [62], in patients undergoing PD. The NLR was higher in the low BAPWV group than in the high BAPWV group. A multivariate analysis found that the NLR was independently associated with BAPWV, and therefore arterial stiffness, in patients receiving PD (β = 0.33, *p* < 0.01). Lu et al. [63] also identified a high NLR as an independent predictor of arterial stiffness in patients undergoing PD, as measured by carotid-femoral PWV (β = 1.150, *p* < 0.001) and the carotid augmentation index (β = 3.945, *p* < 0.001). Patients with higher NLRs had lower survival rates, confirming the association between a high NLR and cardiovascular and all-cause mortality in patients undergoing PD. An et al. [64] also found that the NLR was a strong predictor of both overall and cardiovascular mortality in patients receiving PD.

The risk factors for stroke include hypertension, hyperlipidemia, and atherosclerosis. Although patients receiving PD have a higher risk of arteriosclerosis, few studies have explored the relationship between the NLR and stroke in patients undergoing PD. A multicenter retrospective study categorized patients into three groups: NLR <2.74, 2.74< NLR <4, and NLR >4. The incidence of first stroke in the NLR >4 group was significantly higher than that in the NLR <2.74 group. A high NLR is an independent risk factor for first stroke in patients undergoing PD (*p* < 0.05) [65] and may have predictive significance for the risk of first stroke in these patients, providing early clinical warning.

### NLR and infection

Infection is a common complication in patients undergoing PD, with peritonitis being the most common. Coagulase-negative *Staphylococcus* species are the predominant causative agents of PD-associated peritonitis (PDAP) [32], with touch contamination being a primary contributing factor [32]. PDAP can limit the use of PD and even cause death [66]. Therefore, predicting the outcome of peritonitis treatment is essential; for this, biomarkers are required that can be measured during the early stages of a peritonitis episode. He et al. [67] conducted a retrospective single-center study to investigate the relationship between the NLR and treatment failure in patients with PDAP and found a linear correlation between a higher NLR (>6.53) at the onset of PDAP and an increased risk of treatment failure. The adjusted odds ratio was 1.82 (95% confidence interval 1.05 ∼ 3.15, *p* < 0.05), indicating that the NLR can provide early warning and therefore improve decision-making for PDAP treatment. Another study also showed the predictive value of the NLR for PDAP treatment failure [68]; patients with an NLR >6.53 had a 3.41-fold increased risk of treatment failure compared to those with an NLR <3.75[68]. Interestingly, the NLR could also differentiate between *Mycobacterium tuberculosis* and non-tuberculous mycobacteria in bacterial PDAP [69]. An NLR <15 in the peritoneal dialysate was the optimal cutoff value with a sensitivity, specificity, positive predictive value, and negative predictive value of 81%, 70%, 97%, and 22%, respectively. The NLR may therefore enable the early diagnosis of *Mycobacterium tuberculosis*/non-tuberculous mycobacteria peritonitis, allowing specific analysis and treatment to be initiated earlier. However, a cross-sectional study by Bilen et al. [70] found that the incidence of peritonitis was related to the duration of PD rather than the NLR.

In recent years, the incidence of pneumonia as the first symptom in patients undergoing PD has received increased attention. A Chinese cohort study involving 739 patients undergoing PD found that the high NLR group had a significantly greater incidence of first pneumonia than the low NLR group (*p* < 0.05); patients with a high NLR were at a significantly increased risk of pneumonia. This finding has a certain significance for clinical practice [71].

### NLR and other complications

Mortality associated with PD has been widely studied. In recent years, Xu et al. [72] found that the NLR has prognostic value in patients undergoing PD. An increased NLR (hazard ratio 1.136, 95% confidence interval 1.067–1.210) was a risk factor for all-cause death in these patients. The area under the receiving operator characteristic curve for NLR in predicting all-cause death in patients undergoing PD is 0.698, with a sensitivity of 69.77% and a specificity of 66.78%. Patients with an NLR ≥3.71 have a significantly lower cumulative survival rate than those with an NLR <3.71 (logrank 37.551, *p* < 0.01). Liu et al. [73] reported the risk factors affecting mortality in patients undergoing PD at various time points. They revealed that those patients who passed away within the first three months had a higher NLR, and a high NLR (hazard ratio 1.115, *p* < 0.05) served as a significant risk factor for early death. In summary, the NLR is closely correlated with mortality in patients undergoing PD and can predict prognosis. Therefore, in clinical practice, it is imperative to intensively manage patients with an elevated NLR to enhance survival rates in these patients.

Hyporesponsiveness to EPO is an important concern during dialysis. Approximately 5–10% of patients with chronic kidney disease are resistant to EPO to a certain extent [74]. One study [5] found that the NLR was positively correlated with CRP levels and negatively correlated with serum albumin levels, although no significant correlation between the NLR and the EPO resistance index was observed. However, due to the limited sample size of patients undergoing PD in this study, further follow-up investigations are warranted to confirm these findings. It has been additionally found that an elevated NLR at the initiation of PD is associated with an increased risk of technical failure in patients undergoing PD [75].

## Discussion

Most patients receiving long-term dialysis treatment do so in secondary or local hospitals; therefore, it is in these settings that complications occur [76]. Certain disease complications may remain undetected until exacerbation necessitates treatment in specialized medical facilities, leading to escalated healthcare costs. Therefore, the timely detection of early warning signs of potential complications is imperative. To this end, it is necessary to identify biomarkers that possess predictive and prognostic attributes, while also being suitable for implementation in primary care settings. The NLR, a widely acknowledged inflammatory marker, has potential as a predictor of the occurrence and prognosis of various diseases, including cardiovascular disease [6], tumors [77], liver disease [78,79], autoimmune diseases [80], and vascular disease [81]. Compared with conventional biomarkers, the NLR exhibits variations in the early stages of disease, enabling the prompt identification of the body’s inflammatory state [82]. The NLR detection method is straightforward and cost-effective as it eliminates the need for additional blood samples. Moreover, it not only serves as an indicator of inflammation severity but also plays a pivotal role in disease prognosis. The fact that the NLR is a ratio enhances its reliability and consistency compared with any single indicator.

This review explores the predictive value and application of the NLR in cardiovascular events, infection, and other complications arising in patients undergoing HD and PD. Numerous studies have demonstrated the significant benefits of the NLR for disease risk prediction and prognosis. An NLR >3 is associated with an elevated risk of cardiovascular events, infection, and other complications related to dialysis, ultimately leading to a poorer prognosis. Therefore, the NLR can be considered a robust prognostic indicator for evaluating disease severity and mortality. However, it should be noted that there is no universally agreed cutoff that can be implemented across all clinical scenarios. Given that the NLR reflects the equilibrium between acute and chronic infections, as well as adaptive immunity, alterations in the NLR over time may indicate immune system disorders. When interpreting changes in the NLR, it is crucial to consider potential confounding factors such as nutritional status, underlying diseases, and treatment regimens. In clinical practice, physicians should remain vigilant when treating patients with an abnormal NLR, continuously monitor the NLR, and promptly implement appropriate intervention measures to mitigate disease risk. The combined use of CRP levels and the NLR enables a more precise prediction of complication risk and prognosis in patients undergoing HD [83], thereby facilitating early intervention and management to reduce the likelihood of severe complications.

However, there are still some limitations associated with the data reported here, owing to the few relevant clinical studies discussed in this review, the short follow-up time of most studies, the lack of data, and fluctuations in the NLR in patients undergoing dialysis caused by various factors. Additionally, despite its heightened sensitivity, the NLR exhibits limited specificity and has yet to gain widespread recognition as a definitive inflammatory indicator. In terms of the predictive value of the NLR, it is important to consider the potential association between inflammation and malnutrition, as well as protein-energy wasting, with lymphocytes and serum albumin levels allowing evaluation of nutritional status. Furthermore, there is a significant correlation between the NLR and serum albumin levels, and serum albumin demonstrates superior prognostic capability in patients undergoing dialysis [12,84]. The potential effect of this factor on the predictive value of the NLR requires further investigation. As there is some controversy around the use of the NLR, large-scale, multicenter clinical trials are required to confirm its potential clinical application.

In conclusion, we recommend monitoring the NLR in patients at risk of complications to provide an early warning of potential complications. With continuous progress in research, the NLR is expected to become widely used in clinical practice. The combination of the NLR with other measures may mitigate its diagnostic limitations, provide information on disease progression and prognosis in patients undergoing dialysis, and guide clinical management decisions.
